# The Third Cognitive Revolution: The consequences and possibilities for biomedical research

**DOI:** 10.15252/embr.201847647

**Published:** 2019-04-03

**Authors:** James B Hittner, Almira L Hoogesteijn, Jeanne M Fair, Marc HV van Regenmortel, Ariel L Rivas

**Affiliations:** ^1^ Department of Psychology College of Charleston Charleston SC USA; ^2^ Human Ecology Centro de Investigación y de Estudios Avanzados (CINVESTAV) Mérida Yucatán México; ^3^ Biosecurity & Public Health Los Alamos National Laboratory Los Alamos NM USA; ^4^ School of Biotechnology Centre National de la Recherche Scientifique (CNRS) University of Strasbourg Strasbourg France; ^5^ Center for Global Health‐Division of Infectious Diseases School of Medicine University of New Mexico Albuquerque NM USA

**Keywords:** S&S: Careers & Training, S&S: History & Philosophy of Science, S&S: Media & Publishing

## Abstract

The Third Cognitive Revolution poses particular challenges for biomedical research to adopt new knowledge. Interdisciplinary education at all levels would help to address these.

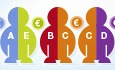

The Third Cognitive Revolution has just started. It follows the ones that, first, brought us the alphabet, numbers, agriculture, and urbanization; and, second, the printing press, books, and the scientific method. The Third Cognitive Revolution (TCR) is characterized by digitalization, computers, the World Wide Web, and global research efforts. While earlier revolutions proceeded at a slow pace over centuries, the current one started only a generation ago and is changing all aspects of human society and even human biology at an unprecedented pace. This leaves little time to analyze the profound effects of these changes and to come to terms with the explosion of knowledge and opportunities that the TCR brings with it. This article explores some of the TCR's positive and some of the troublesome consequences for biomedical research and the social sciences. We focus on two problems: the risk of delaying the adoption of available knowledge and the questionable validity of much of the published literature. To address and hopefully prevent these unintended and problematic developments, we propose and discuss topics that would promote inter‐ and trans‐disciplinary communication.

Generally, the exponential increase of knowledge challenges existing structures that struggle to cope with reviewing and validating it…

While many, if not most, of the TCR's qualities and effects are still poorly understood, some are obvious 1. Perhaps the most relevant aspect was described in 1957 by the economist Robert Solow, who discovered that neither labor nor capital was the critical engine of the economy, but an intangible entity: knowledge 2. While this is good news for science and research, some associated aspects are not.

While earlier revolutions proceeded at a slow pace over centuries, the current one started only a generation ago and is changing all aspects of human society and even human biology at an unprecedented pace.

One problem, described by the communication theorist Everett Rogers as the “diffusion of innovations” challenge, refers to how individuals and institutions react to and adopt new knowledge. Rogers distinguished four categories: innovators, early adopters, late adopters, and non‐adopters. As the late adopters and non‐adopters predominate, the diffusion of innovations problem results in a delay of adopting knowledge that has been produced, paid for, validated, and made publicly available 3. This affects biomedical research in particular where a large percentage of studies devoted to improving human or environmental health is not translated into applicable knowledge. For instance, in spite of about 20,000 articles published on an infectious syndrome (sepsis), the number of novel medical practices based on this knowledge remains close to nil. A similar example is cancer research where < 1% of the published biomarkers have been adopted in clinical practice for diagnostic or therapeutic use.

The peer‐review process has not remained immune to the effects caused by the TCR either. The rapidly growing number of publications including those that cover multiple disciplines has led some to wonder whether journal “editors have the breadth and depth of knowledge or the networks necessary to evaluate all submissions themselves” 4. Generally, the exponential increase of knowledge challenges existing structures that struggle to cope with reviewing and validating it even if digitalization and the Internet now allow for easy, rapid, and global publication. Moreover, the trend for more trans‐ and interdisciplinary research, a direct consequence of the TCR, also challenges review procedures along with the ability of scientists to combine methods and results from seemingly disparate disciplines.

## Easily implemented but invalid: the “streetlight effect”

An additional—and probably larger—problem is the so‐called “streetlight effect” or “tendency for researchers to focus on particular questions, cases, and variables for reasons of convenience or data availability rather than broader relevance, policy import, or construct validity” 5. Also known as the “drunkard search”, this phenomenon is illustrated by an old joke of a person looking for a lost coin, at night, under a streetlight. After learning that the loss occurred far away, a policeman asks the drunkard why he is looking in the wrong site, upon which he replies: “Because the light is better here”. This story describes the concept of construct validity, which, at its core, refers to the following question: do we measure what needs to be measured or do we measure what is easily measured, even if it is invalid or irrelevant 6? When it comes to scientific research, the “streetlight effect” is no longer a joke.

The most abundant manifestation of the streetlight effect is probably adopting a technique or a technology before a question or a problem is formulated.

Examples of the streetlight effect abound in biomedical and social sciences research. One case is the method that has been used over many years to evaluate the efficacy of vaccines against influenza virus. Numerous studies based on measuring immunoglobulin (Ig) G anti‐influenza in blood—which can be easily taken—have missed an important fact: blood IgG correlates poorly with protection. Protection against influenza is mainly conferred by IgA, which is difficult to measure at the location where these immunoglobulins actually act: in the nose.

Another, similar streetlight effect can be found in the history of research on human immunodeficiency virus (HIV). For many years, researchers measured blood CD4^+^ T cells and, given the slow decline of the number of these cells in infected individuals, assumed that the virus replicates slowly. It was only in 1998, when studies showed that HIV—and its related simian immunodeficiency virus, SIV, which infects macaques—rapidly kills huge numbers of intestinal CD4^+^ T cells, while blood T cells remain largely unaffected. Conducting gut biopsies is invasive and very uncomfortable for patients, whereas taking a blood sample is easy. This streetlight effect also had an unfortunate impact on vaccine development: instead of trying to develop injectable vaccines to boost T‐cell levels and other markers in blood, experimenting with oral vaccines to impact T‐cell responses in the gut might have been more relevant.

An additional streetlight effect that resulted in hundreds of studies, at great cost to society, investigated the molecular structure of human monoclonal antibodies that react with HIV. These can be easily measured in peripheral blood 2–3 years after the initial HIV infection, but these antibodies do not protect infected people and cannot be induced by vaccination 7.

## The “solution‐comes‐before‐the‐problem” fallacy

The most abundant manifestation of the streetlight effect is probably adopting a technique or a technology before a question or a problem is formulated. One consequence of this fallacy is the replacement of relevance by the appearance of relevance. Expressions of the “solution‐comes‐before‐the‐problem” fallacy include the uncritical use of *P*‐values as reified decision criteria, that is, the replacement of biological relevance by statistical significance [preprint: 8]. While many scientists and editors seem to believe that small *P*‐values are sufficient to establish scientific progress, such small *P*‐values, in and of themselves, do not necessarily indicate scientific worth—for instance, very large sample sizes result in small standard errors of measurement, which in turn heighten the probability of obtaining small *P‐*values even for small effect sizes. Furthermore, even sophisticated methods for analyzing data cannot fully compensate for inadequate research design. Stated differently, statistical considerations are only pertinent after construct validity—that is, scientific relevance—is established 6, 8.

… the TCR is not just a quantitative, but a qualitative challenge that can promote ignorance.

Moreover, decisions based on *P*‐value thresholds assume that only two outcomes are possible: a finding is either statistically significant or not. Yet, in the real world, more alternatives may exist, which would be missed or confounded by dichotomous paradigms such as *P*‐value thresholding. Therefore, threshold‐based (yes/no or significant/non‐significant) research inquiries are reductionist: they assume that only two alternatives are possible or relevant. While this approach was useful up to three decades ago, reductionist approaches have now been shown to induce errors and omit valuable information 9.

One major example is the medical paradigm “as is our Pathology so is our practice” that was introduced at the end of the 19^th^ century by the physician William Osler. The Oslerian paradigm views diseases as correlations between clinical syndromes and pathological analyses, that is, post‐mortem findings. While it was useful when the optical microscope was the main or only instrument available to analyze tissue samples, the paradigm confuses consequences with causes. Because reductionism does not and cannot examine dynamic and multi‐dimensional processes, it is likely to miss a cause if not observed at post‐mortem inspection. For instance, a bacterial infection of the heart may cause irreversible lesions in the kidney when large immune complexes induced by the original infection get stuck in renal capillaries.

Instead of depending on serendipitous connections across disciplines […], proactive, policy‐driven efforts could be more efficient.

Reductionism also affects pharmacological research: the “target” of a drug may not be a single, distinct, and static site but a dynamic process that involves multiple sites and functions. While recent alternatives derived from General Systems Theory, such as systems biology and systems medicine, attempt to correct the limitations of reductionism, a warning is in order: if such efforts are performed under inadequate interdisciplinary paradigms, they will just perpetuate reductionism.

## The current situation

**Figure 1:**
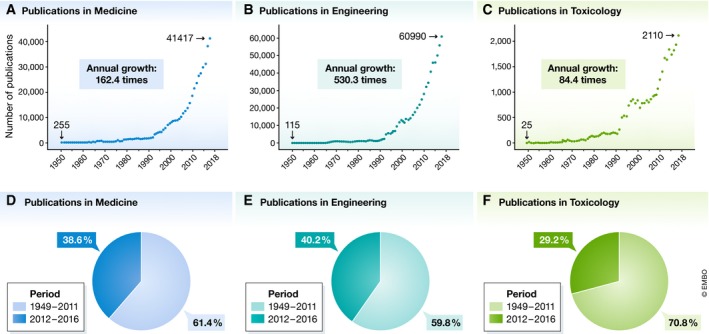
The knowledge explosion The number of publications reported by the *Web of Science*™ under the keywords “medicine”, “engineering”, or “toxicology” is expressed as counts/year (A–C) or percentage of all publications released between 1949 and 2017 (D–F). All investigated fields exhibited an exponential growth (A–C). If, in 1950, the average researcher read 2 h/week to remain updated with the scientific literature, a similar researcher, in 2016, should have read 162.4 × 2 = 324.8 h/week (41,417/255) if his/her field was Medicine (A); 1,060.6 h/week (530.3 × 2) if the area of work was Engineering (B); or 168.8 h/week (84.4 × 2) if involved in Toxicology (C). Knowledge production grows so fast that, in Medicine, 38.6% of all publications generated since 1949 were produced in the last 5 years (D). A similar trend is observed in Engineering and Toxicology, where publications released in the last 5 years represented 40.2 and 29.2% of all works disseminated since 1949, respectively (E, F).

A major hurdle for overcoming these fallacies and reductionist approaches to complex problems comes from the fact that the amount of the available information—and thereby potential information to address a certain problem—far exceeds the human ability to read it (Fig 1A–C). If 2 h/week were enough, in 1955, for the average scientist to become updated with the scientific literature in his/her field, a similar scientist in 2016 would have needed between 308 and 638 reading hours/week and even more in the near future. Yet, a week is just 168 h long.

During the past six decades, the amount of knowledge has “exploded”: the number of publications has increased more than 100‐fold in several fields and continues to grow at a rate of 10% per year. At least ¼ of everything published in science throughout human history was released in the past 5 years (Fig 1D–F). Even assuming a high number of redundant publications, the size of the literature is colossal. While the production of knowledge has been growing since the Middle Ages, the difference between earlier eras and the current one is that now, for the first time in human history, the magnitude and diversity of the available literature exceeds the ability of any human to both read and understand it, which contributes to the diffusion of knowledge challenge described above. Consequently, the TCR is not just a quantitative, but a qualitative challenge that can promote ignorance. That is so because no single person can be aware of, or anticipate even a minor percentage of all hypotheses that can be generated from the available information.

To overcome this combinatorial ignorance, we would need not to know everything (clearly an impossible task) but, instead, to develop a new communication process. While new technologies and self‐learning systems may ameliorate this situation, there is a need for incentives, services, and educational programs that support and accelerate the adoption of information. We need novel procedures that facilitate connections across diverse disciplines; integrate and synthesize knowledge; and identify—as early as possible—new concepts that are likely to influence many fields. Because this situation has never been experienced by any other human generation, no precedents are available. Novel solutions are needed, including: new policies that promote double disciplinary backgrounds; continuing evaluations of academic and publishing institutions; and educational programs based on constant evaluation.

## Double disciplinary backgrounds

A double disciplinary background could improve communications across different fields, generating a combinatorial language and, thereby, yielding new insights, hypotheses, or inspiration for research or products. While defining “double background” may be tricky, examples of that phrase could be training in “statistics and psychology”, “immunology and philosophy of science”, or “ornithology and toxicology”.

While not all possible combinations are likely to generate cognitive breakthroughs, some combinations of apparently unrelated fields might. For instance, could training in Art History, Medicine, and Psychology lead to practical discoveries? We know from Art History that during the Renaissance, artists and scientists, such as Brunelleschi and Leonardo da Vinci, discovered and applied the concept of perspective: how to generate the illusion of a three‐dimensional space on a flat surface. Four centuries later, perspective became a central concept of Gestalt Psychology. Almost another century later, in 1995, the application of Gestalt concepts in embryonic development was rewarded with the Nobel Prize in Medicine for Eric F. Wieschaus, Edward B. Lewis, and Christiane Nüsslein‐Volhard. This example reveals that active cognitive translation is needed, not only to promote communications across fields (and so initiate new or better knowledge) but also to avoid delaying the use of valid knowledge. Instead of depending on serendipitous connections across disciplines (as illustrated by the five century‐long process involved in understanding pattern recognition), proactive, policy‐driven efforts could be more efficient.

Another example of interdisciplinary curricular and institutional changes involves Global Health and the fight against endemic diseases. Some of these could be prevented more rapidly, effectively, and at lower costs, using interdisciplinary approaches that integrate geographical information systems (GIS), human and veterinary medicine, economics, computational sciences/epidemiology (network analysis), and social sciences—especially, communication sciences. Instead, health policies that originated before the emergence of GIS are still being applied even though they are based on erroneous assumptions and, consequently, are costly and ineffective. For instance, vaccination strategies many times lack data on the local biogeography and assume that geography and populations are homogeneous and static. In contrast, the production of high‐resolution maps, together with updated economic and epidemiological considerations, could indicate where and when it is best to vaccinate.

A third example of interdisciplinary combinations—with worldwide applicability—would be courses and research on biologically grounded research methods. While research methodologies are already covered by the current curricula, they do not always investigate the explicit and implicit assumptions of biomedical sciences—also known as Philosophy of Science. Systematic paradigm research to explicitly identify the fundamental assumptions of medical practices or research projects could be the linchpin that connects educational and research activities. To assure that theory and practice would be integrated, such a foundational coursework of doctoral and professional programs should include biomedically relevant examples. Here, interdisciplinary course development is the key concept. The focus would not be the endpoint or course delivery—an individual instructor sharing his/her personal knowledge on a subject matter—but the process of course development, that is, the time and resources needed to advance interactions among methodologists and subject specialists.

**Figure 2:**
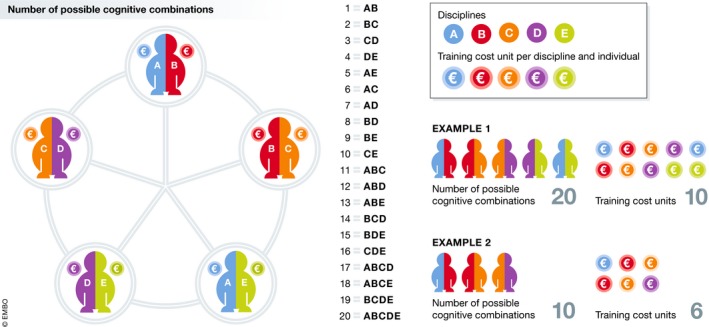
The benefit/cost ratio of trans‐disciplinary translations based on double training Five circles represent five individuals (AB, BC,…AE), who possess a double training. They cover a total of five disciplines (A, B,…E). Because each discipline is shared by two individuals, communications across different disciplines are facilitated by people familiar with the concepts and lexicons of two fields. Such translations induce new disciplinary configurations, which exceed the number of primary (input) disciplines while costs decrease. In this example, the number of possible *cognitive combinations is 20*: (1) AB, (2) BC, (3) CD, (4) DE, (5) AE, as well as (6) AC (links between AB and BC), (7) AD (links between AB, BC, and CD), (8) BD (links between BC and CD), (9) BE (links between BC, AB, and AE), (10) CE (links between CD and DE), (11) ABC (links between AB and BC), (12) ABD (links between AB, BC and CD), (13) ABE (links between AB and DE), (14) BCD (links between BC and CD), (15) BDE (links between BC, CD, and AE), (16) CDE (links between CD and DE), (17) ABCD (links between AB, BC, and CD), (18) ABCE (links between AB, BC, and DE), (19) BCDE (links between BC, CD, and DE), and (20) ABCDE (links between AB, BC, CD, and DE). Assuming that double training costs twice higher than average (2 cost units/individual), *the training cost* for 5 individuals is 10 units. Therefore, in this example, the benefit/cost ratio (20/10) is equal to 2. If, instead, only the first three individuals were considered (AB, BC, and CD), who covered four disciplines (A, B, C, D), the number of possible *cognitive combinations would be 10*: (1) AB, (2) BC, (3) CD, as well as (4) AC (links between AB and BC), (5) AD (links between AB, BC, and CD), (6) BD (links between BC and CD), (7) ABC (links between AB and BC), (8) ABD (links between AB, BC, and CD), (9) BCD (links between BC and CD), and (10) ABCD (links between AB, BC, and CD). Considering the same assumptions (double training costs twice higher than average), the *training cost for 3 individuals would be 6 units*. Thus, in the second example, the benefit/cost ratio would be (10/6) 1.67. Therefore, a 67% ([5–3]/3 or 2/3) increase in doubly trained personnel—example 1 minus example 2—results in a 100% (20/10) increase in combinations and a 33% increase in the benefit/cost ratio. In other words, the larger the number of disciplinary combinations, the lower the cost of interdisciplinary translations, and the greater the benefit/cost ratio. Designs that promote understanding across fields are both needed and more economical than alternatives.

Would such interdisciplinary combinations be too costly? Evidence suggests that the larger the proportion of faculty members with a double training—a requisite for successful translations across fields—the larger the benefits and the smaller the costs (Fig 2).

## Continuous evaluations

The efficient translation of knowledge depends on evaluations that involve not only individual persons but also curricula, structures, or decisions produced by or affecting academia and scientific publications. Such evaluations could start as early as possible—before any project is conceived—and finish as late as possible. Only through permanent knowledge construction, de‐construction and re‐construction, can errors and omissions be identified and, hopefully, corrected. To that end, judging the past (“summative” evaluations) should not be the priority but, instead, processes and interactions that shape the future (“formative” evaluations) should be prioritized. Evaluations also are needed to prevent asymmetric relationships: unbalanced or unidirectional flows that benefit one discipline or field at the expense of another.

While most universities created in the 19^th^ century served the needs of the industrial era (when specialization was needed), the digital age is based on information and knowledge.

Given their central role in evaluating and disseminating knowledge, scientific publications should also adapt to the challenges of the TCR, notably by offering new services to enable early detection, synthesis, and dissemination of novel concepts; promulgating policies that explicitly prevent fallacies and promote inter/trans‐disciplinary synthesis; and enhancing the education of journal reviewers. Examples of new services would include bibliographic analyses to detect or compare topics with a rapid growth of citations, identifying concepts that are cited in a different field or discipline, and highlighting cognitive contents within similar disciplines, such as human, veterinary and plant microbiology. An example of journal policies is “research on published research” to document fallacies, omissions, and/or tacit but not demonstrated assumptions in published manuscripts 10. Such policies should foster inter‐/trans‐disciplinary translations—not simple multi‐disciplinary connections. This distinction matters because mixing differs from integrating. While multi‐disciplinary expertise only involves cognitive juxtaposition or the use of pre‐established knowledge, inter/transdisciplinarity integrates or creates new, usually need‐specific, knowledge.

Together, these policies and changes across academia and scientific publishing should help to unravel the Gordian knot of the Third Cognitive Revolution: the peer‐review process which, many times, evaluates knowledge without formal training, without evaluation of the quality of the review, without supervision, without legal responsibilities, and without rewards 10. While most peer‐reviewers are correctly chosen for their expertise, many lack adequate training in statistics, research methodology, or other fields that are necessary to better judge the quality of much of biomedical research. Such training deficits have caused errors and deficiencies, which, at least partially, may explain “streetlight effects” and published but unusable knowledge. It is not just reviewers but also editors, authors, and graduate students who require new educational programs that emphasize both communication and methodological skills.

These remedies might help both Academia and scientific publishing to renew and adapt to the challenges of the TCR. While most universities created in the 19^th^ century served the needs of the industrial era (when specialization was needed), the digital age is based on information and knowledge. The current priority for education and evaluation is, therefore, not only specialized knowledge, but broader skills that enrich cognitive growth and integrated (interdisciplinary) knowledge so “streetlight effects” can be prevented 1. While the earlier cognitive revolutions took place mostly spontaneously and at a leisurely pace, the Third Cognitive Revolution offers—for the first time in history—the opportunity to use the available knowledge to steer and guide its translation and adaptation.

**Further reading**

Cohen J, Vincent JL, Adhikari NKJ, Machado FR, Angus DC, Calandra T, Jaton K, Giulieri S, Delaloye J, Opal S *et al* (2015) Sepsis: a roadmap for future research. *Lancet Infect Dis* 15: 581–614

Kern SE (2012) Why your new cancer biomarker may never work: recurrent patterns and remarkable diversity in biomarker failures. *Cancer Res* 72: 6097–6101

Yordanov Y, Dechartres A, Porcher R, Boutron LA, Altman DG, Ravaud P (2015) Avoidable waste of research related to inadequate methods in clinical trials. *BMJ* 350: h809

Battaglia M, Atkinson MA (2015) The streetlight effect in type 1 diabetes. *Diabetes* 64: 1081–1090

Maurer MA, Meyer L, Bianchi M, Turner HL, Le NPL, Steck M, Wyrzucki A, Orlowski V, Ward AB, Crispin M *et al* (2018) Glycosylation of human IgA directly inhibits influenza A and other sialic‐acid‐binding viruses. *Cell Rep* 23: 90–99

Veazey RS, Lackner AA (2004) Getting to the guts of HIV Pathogenesis. *J Exp Med* 200: 697–700

Van Regenmortel MHV (2015) Paradigm changes are required in HIV vaccine research. *Front Immunol* 6: 326

Loscalzo J, Barabasi A‐L (2011) Systems biology and the future of medicine. *Wiley Interdiscip Rev Syst Biol Med* 3: 619–627

Earm K, Earm YE (2014) Integrative approach in the era of failing drug discovery and development. *Integr Med Res* 3: 211–216

Saetzler K, Sonnenschein C, Soto AM (2011) Systems biology beyond networks: Generating order from disorder through self‐organization. *Sem Cancer Biol* 21: 165–174

Weissmann G (2010) Pattern recognition and gestalt psychology: the day Nusslein‐Volhard shouted “Toll!”. *FASEB J* 24: 2137–2141

Cassi L, Champeimont R, Mescheba W, de Turckheim É (2017). Analysing institutions interdisciplinarity by extensive use of Rao‐Stirling Diversity Index. *PLoS One* 12: e0170296

Bärnighausen T, Bloom DE, Cafiero‐Fonseca ET, O'Brien J (2014) Valuing vaccination. *Proc Natl Acad Sci USA* 111: 12313–12319

Rivas AL, Fasina FO, Hammond JM, Smith SD, Hoogesteijn AL, Febles AL, Hittner JB, Perkins DJ (2012) Epidemic protection zones: centred on cases or based on connectivity? *Transb Emerg Dis* 59: 464–469
